# Mechanical Positive Pressure Ventilation and Voice Training via the Blom® Tracheostomy Tube: A Case Study

**DOI:** 10.7759/cureus.36375

**Published:** 2023-03-19

**Authors:** Tomohide Takei, Tatsuya Kida, Yutaka Usuda

**Affiliations:** 1 Anesthesiology, Yokohama City University Medical Center, Yokohama, JPN; 2 Anesthesiology, Yokosuka Kyōsai Hospital, Yokosuka, JPN; 3 Intensive Care Unit, Yokosuka Kyōsai Hospital, Yokosuka, JPN

**Keywords:** speech cannula, positive end-expiratory pressure, vocalization, tracheostomy tube, mechanical ventilation

## Abstract

Patients with a Blom® tracheostomy tube (containing a cuff) can vocalize while on mechanical ventilation, which can significantly improve the patient's quality of life. This is brought by the purpose-built structure of the tracheostomy tube that allows the expiration to be expelled through the glottis. However, this characteristic may complicate the measurement of the patient's tidal volume, as most of the expiration does not return to the ventilator. Owing to the necessity of insertion of the speech cannula, which acts as an inner cannula, to enable patient vocalization, the air passage likely becomes constricted, thus increasing airway resistance. Difficulty in applying appropriate positive end-expiratory pressure (PEEP) and ventilator auto-triggering may also be problematic. Therefore, alveolar ventilation is predicted to decrease without adjusting the ventilation settings. Our experience using the Blom® tracheostomy tube revealed some problems, and we provide suggestions for patient management. We herein report on the experience of a patient having inserted the Blom® tracheostomy tube receiving mechanical positive pressure ventilation during vocal training.

## Introduction

Patients receiving mechanical ventilation cannot speak, which greatly impairs their quality of life. Since Safar P and Grenvik A [1] described a speech cannula that enables vocalization via inhaled gas passing through the patient's glottis, various types of speech cannulas have been devised and improved. However, only a few commercially available cannulas enable speech and positive pressure ventilation. Blom® (Pulmodyne; Indianapolis, Indiana, USA) is one such product [2]. As it has only been available since December 2011, few reports exist on its use. Several studies have reported their experience with the product representing the improved quality of vocalizing [3]. However, to our knowledge, no studies have addressed the consideration of various ventilation parameters during the use of this product. Herein, we report various ventilation parameters of a patient receiving ventilation with this product and discuss the results in terms of advantages and necessary precautions. The Blom® tracheostomy tube enables vocalization along with simultaneous positive pressure ventilation by inserting a speech cannula as an inner tube into the outer cannula. The tip of the speech cannula is shaped as a one-way valve, with a fenestration located just above the cuff so that the exhaled air passes through the tube in the direction of the glottis as the one-way valve closes [3]. It can also be used as a conventional tracheostomy tube by replacing the inner tube with a standard cannula or a suction cannula.

## Case presentation

**Figure 1:**
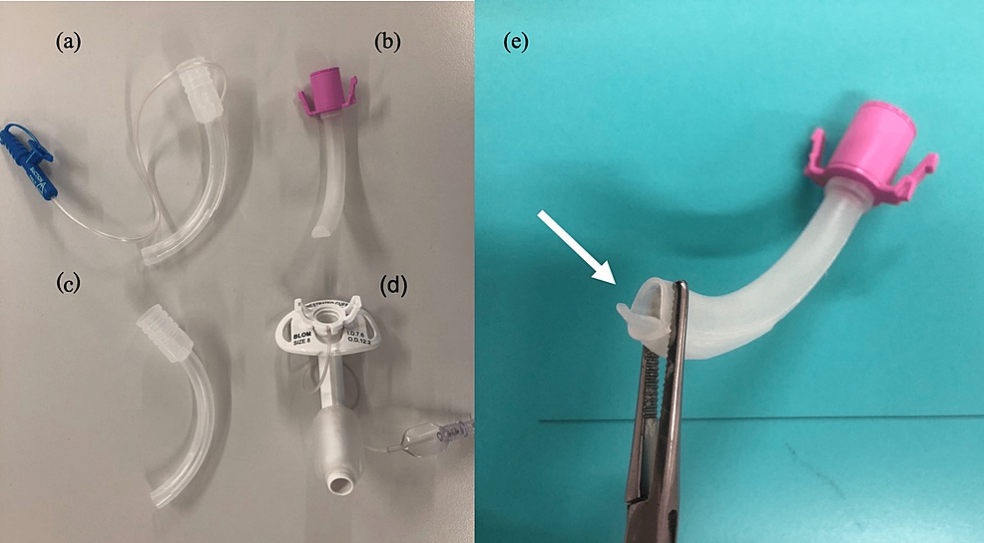
The Blom® tracheostomy tube with the different cannulas used separately. (a) Suction cannula; (b) Speech cannula; (c) Standard cannula; (d) Outer cannula; (e) Tip of speech cannula; the white arrow points out the one-way valve.

 A 66-year-old male patient with a height and weight of 160 cm and 60 kg, respectively, was admitted to the ICU due to rapidly progressive quadriplegia and dyspnea. A mass lesion was detected extending from the right side of the third cervical vertebra to the right brachial plexus on CT, followed by a biopsy, revealing the diagnosis of malignant lymphoma (diffuse large B-cell lymphoma [DLBCL]). Emergency tracheal intubation and ventilatory management were established due to respiratory failure caused by bilateral cervical spinal cord impairment at the C4 level. Tracheostomy was performed along with chemotherapy, as prolonged ventilation was expected. A Blueline Ultra® Suction-Aid with a cuff (PORTEX®; Smiths Medical, Ashford, Kent, UK) was used for the tracheostomy tube. However, as the patient became conscious and clear, he began to complain of being unable to communicate with medical staff members. Thus, after consulting with the attending physician and the patient, we decided to insert the Blom® tracheostomy tube. It was thought that enabling the patient to speak would reduce stress on both the patient and the medical staff. The ventilator used was VELA® (Vyaire Medical, Mettawa, Illinois, USA), and all the parameters described below were obtained from the same patient. Soon after the insertion of Blom® tracheostomy tube #8 (OD 12.2 mm, ID 7.6 mm), the patient was able to vocalize clearly. We decided to start with a duration of 10 minutes of vocal training per day. The ventilator settings were adjusted (see below) during vocal training to maintain sufficient alveolar ventilation. In addition, except during training (normal state), a suction cannula inner tube that allowed positive pressure ventilation along with suction over the cuff was inserted into the cannula instead of the speech cannula (Figure 1).

During vocal training, the pressure control-assist/control (PC-A/C) mode controlled ventilation throughout the day. Ventilator parameters were frequency (12 per minute), maximum inspiratory pressure (12 cmH2O), positive end-expiratory pressure (8 cmH_2_O), inspiratory time (1.2 seconds), flow trigger (2 L per second), and F_I_O_2_ (0.25). During vocal training, we adjusted the frequency from 12 to 20 per minute and the maximum inspiratory pressure from 12 to 22 cmH2O and increased the FIO2 to 0.5. Both the inspiratory and expiratory tidal volumes were approximately 500 mL in the normal state. However, during vocal training, the inspiratory tidal volume was 500 mL on the ventilator monitor, and the expiratory tidal volume was approximately 90 mL, indicating a discrepancy. The blood gas analysis results at both time points are listed in Table 1. Vocal training was discontinued if the patient complained of significant discomfort or fatigue, if hypoxemia developed, or if tachypnea or other symptoms were observed.

**Table 1:** Ventilator settings with/without vocal training and the results from the blood gas analysis.

	Normal state	Vocal training
Ventilator settings		
Mode	PC-A/C	PC-A/C
Inspiratory pressure (cmH_2_O)	12	22
Respiratory frequency (per min)	12	20
Positive end expiratory pressure (cmH_2_O)	8	8
F_I_O_2_	0.25	0.5
Blood gas analysis		
pH	7.438	7.363
pO_2_ (Torr)	94.7	135
P/F ratio	388.8	270
pCO_2_ (Torr)	41.2	50.7
pHCO_3_^-^ (Torr)	27.4	28.1
Expiratory Tidal Volume (ml)	482	95
Inspiratory Tidal Volume (ml)	478	502
Pressure Control-Assist/Control (PC-A/C)		

Vocal training and mechanical ventilation were practiced continuously until the patient was discharged from the ICU.

## Discussion

In this case, the insertion of the Blom® tracheostomy tube speech cannula enabled the patient to speak. This product enables vocalization while providing positive pressure ventilation, which was previously thought to be impossible. However, the product has several limitations. First, depending on the ventilator, the tidal volume measurement is usually shown on the monitor during expiration. Therefore, when this product is inserted and mechanical ventilation is performed, it may be difficult to accurately measure the tidal volume by observation alone because much of the expiration is expelled through the glottis and not returned to the ventilator. This can be handled by changing the measurement of the tidal volume displayed on the monitor to that of inspiration; however, it should be noted that, in this case, it is still a challenge to judge whether ventilation is being properly performed only by watching the monitor. It should also be noted that the lower-minute ventilation volume limit alarms in many ventilators to continue to operate improperly.

Therefore, positive end-expiratory pressure (PEEP) may not be properly applied to the patient. During exhalation, the one-way valve at the end of the speech cannula shown in the figure above closes, allowing the patient to freely relieve pressure in the airway through the nasal orifice until the next inhalation is initiated either by forced exhalation or according to the patient's lung thorax compliance. At this time, the ventilator monitor does not reflect this decrease in airway pressure because the ventilatory circuit becomes a closed circuit by the closed one-way valve. Therefore, caution should be exercised in its use in patients with conditions requiring a high PEEP. In fact, in the present case, a decrease in the P/F ratio was observed in the absence of a decrease in the minute volume after the insertion of the speech cannula. Furthermore, inserting a speech cannula may increase airway resistance by narrowing the tracheostomy tube lumen. The resistance value at the time of speech cannula insertion, which was simply measured using an artificial lung at our hospital, was 0.33 cmH_2_O/L/min, which roughly approximated the resistance value when forced ventilation was performed by intubating a tracheal tube with an inner diameter of 6.0 mm [4]. In our case, ventilatory management was performed using the PC-A/C. After insertion of the speech cannula, inspiratory pressure should be adjusted to prevent the minute volume rate from decreasing. Volume control-assist/control (VC-A/C) ventilation can be a solution, but it can lead to an unexpected increase in inspiratory pressure, and synchronization with patient inspiration can also be problematic [5]. Therefore, the use of different ventilation modes, depending on the situation, should be considered. In addition, auto-triggering can be problematic. When the exhalation time is too long in the ventilator setting, the airway pressure decreases by air escaping, as described above. When it falls below the set PEEP value, inspiratory flow is generated, leading to unexpected triggering. This occurs even during vocal training and can not only interfere with speech but also cause discomfort to the patient. Based on the facts above, inspiratory pressure should be increased, and expiratory time should be shortened during vocal training. As all the measurements were obtained from the same patient, it is unclear whether our ventilator re-settings can be applied to other patients. Although, some previous papers have also recommended increasing the maximum inspiratory pressure to 10 cmH_2_0, which is similar to our trial [3]. Further research and reports are awaited for more firm respiratory management.

## Conclusions

With the advent of the Blom® tracheostomy tube, patients who had difficulty weaning themselves from mechanical ventilation were able to speak, and their quality of life was thought to have improved greatly. However, the actual use of the device revealed that several limitations remain regarding the usage of the product, such as difficulty in measuring ventilatory parameters, applying appropriate PEEP, increasing airway resistance, and occurrence of auto-triggering, which requires proper management and understanding. Due to the different structure from conventional tracheostomy tubes, one must familiarize oneself with its features and properties to handle it appropriately and maximize patient comfort.
